# On the Role of Electrostatic Repulsion in Topological Defect-Driven Membrane Fission

**DOI:** 10.3390/membranes11110812

**Published:** 2021-10-25

**Authors:** Ekaterina Gongadze, Luka Mesarec, Samo Kralj, Veronika Kralj-Iglič, Aleš Iglič

**Affiliations:** 1 Laboratory of Physics, Faculty of Electrical Engineering, University of Ljubljana, 1000 Ljubljana, Slovenia; ekaterina.gongadze@fe.uni-lj.si (E.G.); mesarec.luka@gmail.com (L.M.); 2 Condensed Matter Physics Department, Jožef Stefan Institute, Jamova 39, 1000 Ljubljana, Slovenia; samo.kralj@um.si; 3 Faculty of Natural Sciences and Mathematics, University of Maribor, Koroška 160, 2000 Maribor, Slovenia; 4 Laboratory of Clinical Biophysics, Faculty of Health Sciences, University of Ljubljana, 1000 Ljubljana, Slovenia; kraljiglic@gmail.com; 5 Faculty of Medicine, University of Ljubljana, 1000 Ljubljana, Slovenia

**Keywords:** fission of vesicles, electric double layer, osmotic pressure, orientational ordering of water dipoles, topological defects

## Abstract

Within a modified Langevin Poisson–Boltzmann model of electric double layers, we derived an analytical expression for osmotic pressure between two charged surfaces. The orientational ordering of the water dipoles as well as the space dependencies of electric potentials, electric fields, and osmotic pressure between two charged spheres were taken into account in the model. Thus, we were able to capture the interaction between the parent cell and connected daughter vesicle or the interactions between neighbouring beads in necklace-like membrane protrusions. The predicted repulsion between them can facilitate the topological antidefect-driven fission of membrane daughter vesicles and the fission of beads of undulated membrane protrusions.

## 1. Introduction

The main building block of biological membranes is the lipid bilayer, with embedded inclusions such as proteins [1]. Isotropic and anisotropic membrane proteins may induce local changes in the membrane curvature [2,3], which may result in global changes in the cell shape [4,5,6,7,8,9,10]. The non-homogeneous lateral distribution and the phase separation of membrane inclusions (nanodomains) are the driving forces for transformations of the cell shape [4,5,6,7,10,11,12,13,14,15]. The biological and lipid membranes also possess certain degrees of in-plane orientational ordering [2,5,6,8,16,17,18,19], including nematic type of ordering [20,21,22], which is important for the stability of different membrane shapes. The focus of this article is the role of electric double-layer electrostatics interaction in membrane fission driven by topological antidefects in thin membrane necks.

Topological defects (TDs) appear in biological membranes if they exhibit in-plane membrane orders (e.g., due to anisotropic membrane constituents [2,5,23], collectively tilted flexible hydrocarbon chains [2,20,21,24], or embedded or attached anisotropic membrane constituents [2,5,6]). Their number and positions are in general robustly governed by the topology and geometry [2,3,18,23,25].

Membranes that exhibit in-plane orders are in the first approximation treated as effectively two-dimensional (2D) curved and ordered manifolds. A vast majority of theoretical and numerical studies of TDs in such environments have been realised in 2D models and nematic liquid crystalline (LC) shells [25,26,27,28,29]. TDs in such systems are characterised by the winding number m, which quantifies the total rotation of the relevant orientational field divided by 2π, encircling the core of the defect counter clockwise, and is a discrete and conserved quantity. TDs bearing positive or negative signs of m are commonly referred to as defects or antidefects, respectively. The total value, $m_{\mathrm{tot}}$ of m, on closed surfaces is determined by the Gauss–Bonnet theorem. For the spherical topology of manifolds, $m_{\mathrm{tot}}=2$ holds. Therefore, the surface curvature efficiently stabilises TDs, where patches exhibiting positive (negative) Gaussian curvature attract defects (antidefects) [26,27,30]. Topological defects and antidefects may play important roles in determining the cell membrane shape and membrane fission, as discussed in the following section.

## 2. Topological Defects/Antidefects and Membrane Fission

In the case of artificial lipid and biological membranes, topological defects often occur in thin membrane necks due to a strong negative Gaussian curvature in the neck region.

**Figure 1:**
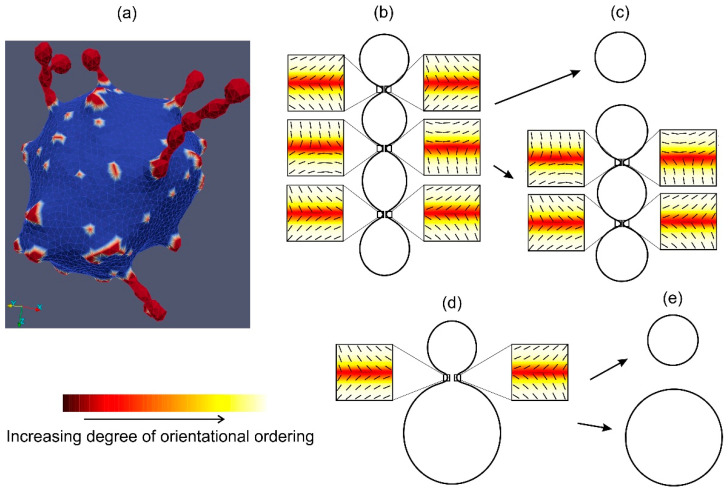
Typical vesicle shapes calculated by MC simulations [11] for the two-component membrane composed of highly curved isotropic flexible nanodomains (marked in red) and the nanodomains with zero intrinsic curvature (marked in blue) are presented in panel (**a**). Membrane nanodomains with high intrinsic curvature (red) are accumulated in undulated membrane protrusions. Panels (**b**,**c**) show the orientational ordering profiles in the necks of undulated membrane buds/protrusions. Topological antidefects are accumulated in the necks. Consequently, the shape with three prominent thin necks (**b**) is transformed into two distinct closed membrane shapes (**c**) as a result of the rupture of one neck. The positions of antidefects in panels (**b**,**c**) are marked by small squares. Orientational ordering profiles with the superimposed nematic director fields in the vicinity of topological antidefects are magnified. The figure also shows an example of the vesicle budding (panel (**d**)) and the formation of the detached daughter vesicle (panel (**e**)) driven by the formation of topological antidefects in the neck prior to the fission process. The shape and orientational ordering profile were calculated as described in [22]. Panels (**a**–**c**) are adapted from [11,22].

As an example, Figure 1a shows a non-axisymmetric vesicle shape [11] that may possess a nematic LC order in the necks of undulated protrusions [22,31]. In panels (b–d) of Figure 1, nematic orientational ordering profiles in the neck regions of necklace-like buds/endovesicles, are presented. The colour plot represents the degree of nematic ordering, while the nematic director field (i.e., the orientation of molecules) is denoted by thin lines. In our visualisation, the light yellow colour represents a high degree of orientational order. At the core of topological defects/antidefects, the nematic order is lost [25,32,33]. Therefore, topological defects/antidefects are located at the surface patches with the lowest degree of orientational ordering, which are marked by dark red in Figure 1. The approximate positions of topological antidefects in thin membrane necks are schematically shown in panels (b–d) of Figure 1 and marked by small squares. The orientational ordering profiles in the vicinity of topological antidefects are magnified [19].

Note that topological defects/antidefects are a source of large local elastic penalties. Consequently, at the cores of TDs, the ordering field is essentially melted [34,35,36,37] (i.e., the degree of nematic ordering is relatively weak). In Figure 1b,d, two antidefects are located within each neck on a relatively small surface area. For this reason, the local interactions between the neighbouring molecules within the neck regions (there, the concentration of antidefects is relatively high) are weakened, which might result in the neck rupture, leading to the fission process [34,38,39]. This process is shown in Figure 1c,e, where two distinct closed membrane shapes (vesicles) are formed. Panel (d) in Figure 1 shows the budding of the parent vesicle and the formation of the single smaller daughter vesicle, which can be detached from the parent vesicle due to neck rupture driven by topological antidefects in the neck. Note that there is no need for antidefects after the fission process because no neck with strong negative Gaussian curvature (i.e., a large curvature deviator) exists [22,26].

Note that, in addition to the fission, TDs in membranes could play important roles in several other mechanisms. For example, they could provide attractive sites for appropriate nanoparticles, nanovesicles, or extracellular vesicles [4]. Moreover, regions hosting TDs might be exploited for cross-membrane transport because intermolecular binding in the corresponding region is weaker. Furthermore, pioneering studies reveal that dynamical vortices (i.e., topological defects in a velocity field) within membrane plasma might play a role in mitosis [40,41]. TDs might present a region of entry for nanoparticles (NPs) in lipid membranes. In general, the biological interaction mechanism of NPs and nanovesicles with cell membranes containing topological defects is not well understood [42].

Structures rich in TDs could be also stabilised or induced by appropriate NPs or extracellular vesicles [4], and other types of membrane nanovesicles. NPs introduce TDs in qualitatively two different ways depending on the strength of their interaction with the surrounding medium possessing orientational ordering. In the case of sufficiently weak interactions [43,44], they tend to assemble within cores of TDs. Consequently, they partially relieve free energy penalties introduced by TDs due to the Defect Core Replacement (DCR) mechanism [44]. Namely, cores of TDs are in general energetically expansive. If cores of TDs are partially replaced by volume of NPs, the relatively high-energy penalty of TDs is reduced. For strong enough interactions, NPs could effectively act as TDs because the NPs’ shapes introduce curvature into the system. Due to the topological charge conservation law, additional TDs are formed in the enclosing medium possessing orientational ordering [45].

It can therefore be assumed that membrane topological defects could be favourable points for their interactions with nanoparticles, extracellular vesicles, and membrane nanovesicles. Hence, in the future, one of the major goal of the research connected to cell membrane physics will be to gain a deeper understanding into the mechanisms of interactions of NPs, nanovesicles, and extracellular vesicles with the cell membrane mediated by topological defects in the membrane regions that possess an orientational order.

Regarding membrane budding and fission, it is shown in this paper that, in addition to topological defects, the electrostatic forces may facilitate membrane fission. Therefore, in the following, we describe the possible role of electrostatic interactions in the membrane fission.

## 3. Electrostatic Interaction between Charged Membrane Surfaces

**Figure 2:**
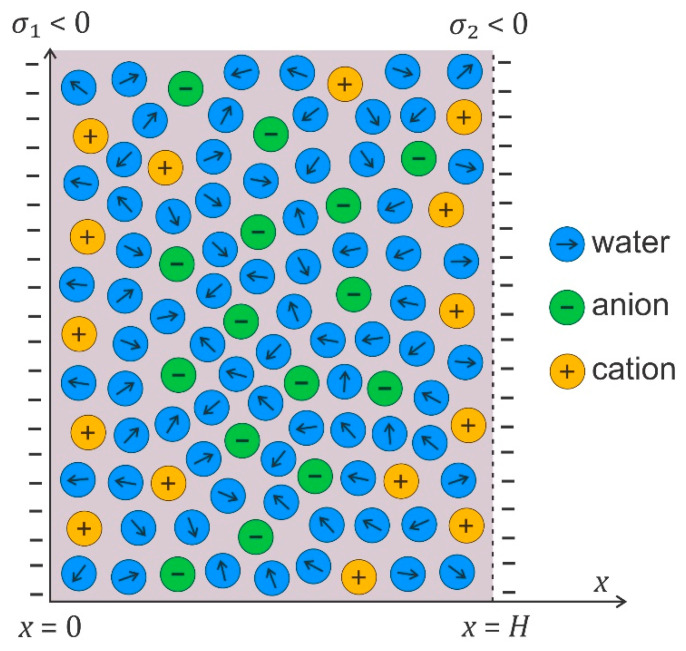
A schematic figure of the electrolyte solution between two charged surfaces at the distance H. The surface charge densities of both surfaces are negative, $\sigma_{1}<0$ and $\sigma_{2}<0$.

Electrostatic interactions between the charged surface and electrolyte solution result in the formation of an electric double layer (EDL) near the charged surface [46,47,48,49,50,51,52,53,54,55]. In an EDL, the ions with electric charges of the opposite sign than the charged surface (counterions) are accumulated close to the charged surface and the ions with a charge of the same sign as the surface (co-ions) are depleted from this region [46,47,48,56,57,58,59]. Figure 2 presents the electrolyte solution between two charged surfaces with surface charge densities of opposite signs, where EDLs are created at both charged surfaces. Due to the non-homogeneous distribution of ions in EDLs, the electric field strength is screened at larger distances from the charged surface. The water dipoles are strongly oriented in a strong electric field of the EDL near the charged surface (Figure 2) [2,55,60,61,62,63,64,65,66,67,68].

In the past, the first theoretical description of EDL was introduced by Helmholtz [69,70], who assumed that a single layer of counterions forms at the charged surface. Later, the spatial distribution of point-like ions in the vicinity of charged surface have been described by the Boltzmann distribution function [46,47]. The finite size of ions in theoretical description of EDL was firstly incorporated by Stern [56] with the so-called distance of closest approach and later developed further by Bikerman, Freise, Eigen, and Wicke [48,57,58,59]. Their work was further improved by numerous theoretical studies and simulations [2,49,50,51,53,62,64,66,68,71,72,73,74,75,76,77,78,79,80,81,82,83,84,85,86,87,88,89,90,91,92]. The physical properties of the EDL are crucial in understanding the interactions between charged membrane surfaces in contact with electrolyte solutions [2,55,93,94,95,96,97,98,99,100,101,102,103].

### 3.1. Modified Langevin Poisson–Boltzmann Model

In the following, we describe the theoretical consideration of electrostatic interactions between charged surfaces, where the orientational degree of freedom of water dipoles is taken into account. Among others, we derive within the modified Langevin Poisson–Boltzmann model [55,104,105] an analytical expression for the osmotic pressure between two charged surfaces (Figure 2), which can be then used for the calculation of net osmotic pressure between two membrane surfaces.

We start with a short description of the modified Langevin Poisson–Boltzmann (LPB) model of an electric double layer [55,104,105], which presents the generalisation of classic Poisson–Boltzmann (PB) theory for point-like ions by taking into account the orientational ordering of water molecules in EDL (see also Figure 2). In the modified LPB model, the orientational ordering of water dipoles is considered close to the saturation regime or in the saturation regime, which leads to the prediction that the relative permittivity close to the charged surface is considerably reduced [55]. The modified LPB model also takes into account the electronic polarisation of the water [55,105]. The space dependency of the relative permittivity within the modified LPB model is given by the following [55,100,105]:

(1) $$\varepsilon_{r}\left(r\right)=n^{2}+\frac{n_{w}p_{0}}{\varepsilon_{0}}\left(\frac{2+n^{2}}{3}\right)\left(\frac{L\left(\gamma p_{0}E\left(r\right)\beta \right)}{E\left(r\right)}\right),$$

which then appears in the modified LPB equation for electric potential ϕ [55,100,105]:

(2) $$\nabla \cdot \left[\varepsilon_{0}\varepsilon_{r}\left(r\right)\nabla \right]=-\rho \left(r\right),$$

where

(3) $$\rho \left(r\right)=e_{0}\;n_{+}\left(r\right)-e_{0}\;n_{-}\left(r\right)=-2e_{0}n_{0}\sin h\left(e_{0}\phi \left(r\right)\beta \right)\;$$

is the macroscopic (net) volume charge density of the electrolyte solution and

(4) $$n_{+}\left(r\right)=n_{0}e^{-e_{0}\phi \left(r\right)\beta }\;,\;n_{-}\left(r\right)=n_{0}e^{e_{0}\phi \left(r\right)\beta }$$

are the number of densities of monovalent cations and anions, respectively. Here, n is the refractive index of water, $n_{0}$ is the bulk number density of ions, $n_{w}$ is the bulk number density of water, and $p_{0}$ is the magnitude of the dipole moment of water molecule. $L\left(u\right)=\coth(u)-1/u$ is the Langevin function; $\gamma =\left(2+n^{2}\right)/2$, $E\left(r\right)$ is the magnitude (absolute value) of the electric field strength; and β=1/kT, where kT is the thermal energy. In the limit of vanishing electric field strength, the above expression for the relative permittivity yields the Onsager limit expression [2,55,60,100]:

(5) $$\varepsilon_{r,b}=n^{2}+{\left(\frac{2+n^{2}}{3}\right)}^{2}\frac{n_{w}p_{0}^{2}\beta }{2\varepsilon_{0}}.$$

at room temperature T=298 K, $p_{0}=3.1$ Debye (the Debye is 3.336 × 10^−30^ C/m), and $n_{w}/N_{A}$ = 55 mol/l, Equation (5) gives $\varepsilon_{r,b}$ = 78.5 for the bulk solution. The value $p_{0}=3.1\;D$ is smaller than the corresponding value in previous similar models of electric double layers considering also orientational ordering of water dipoles. For example, in the model of Abrashkin et al. [106], where the cavity field and electronic polarisability of the water molecules are not taken into account, the value of $p_{0}=4.86\;D$. The model [106] also incorrectly predicts the increase in the relative permittivity of the electrolyte solution in the direction towards the charged surface, which is in contradiction to the experimental results and defies common principles in physics [65,101,104,105]. On the contrary, Equations (1)–(3) of the described modified LPB model predicts the decrease in relative permittivity in the electrolyte solution near the charged surface [2,55,100], in agreement with the experimental observations [107,108].

**Figure 3:**
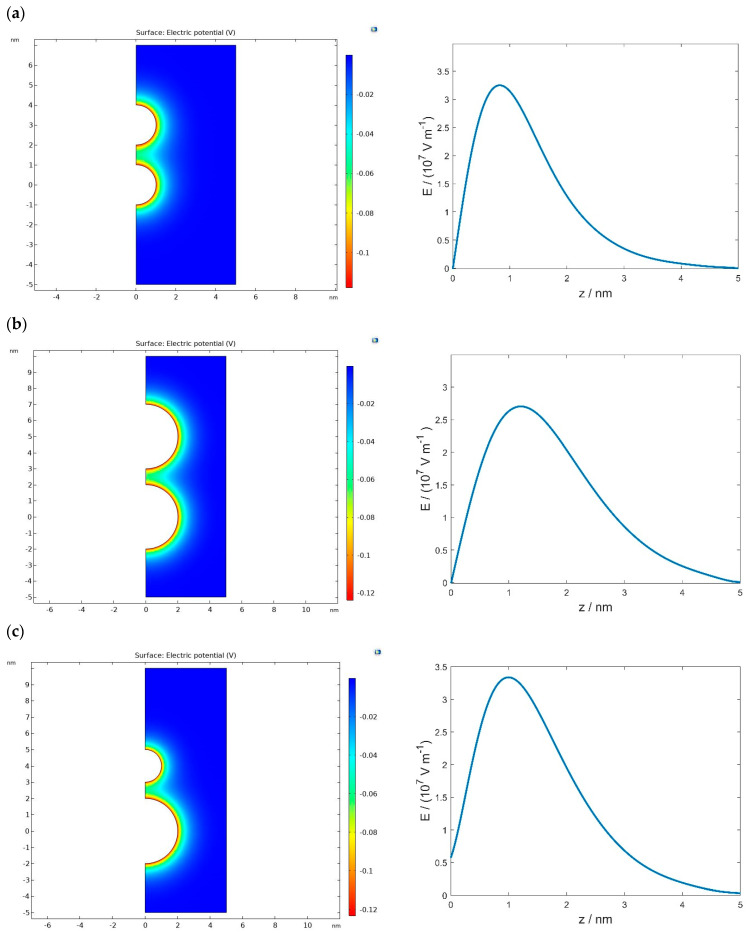
Distribution of the electric potential and the magnitude of the electric field strength along the line passing through the midpoint between the surface of both spheres perpendicular to the line connecting the centres of the sphere (see Figure 4). The calculation were performed by solving the modified LPB eqution (Equations (1)–(3)) for two spheres with equal surface charge densities σ=−0.25 As/m^2^. The radii of the spheres are $R_{p}=R=$ 1 nm (**a**), $R_{p}=R=$ 2 nm (**b**), and $R_{p}=$ 2 nm and R= 1 nm (**c**) (see also Figure 4). The values of other parameters are H=1 nm (see Figure 4), $p_{0}=$ 3.1 Debye, $n_{0}/N_{A}=$ 0.15 mol/L, $n_{w}/N_{A}=$ 55 mol/L, and T= 298 K.

**Figure 4:**
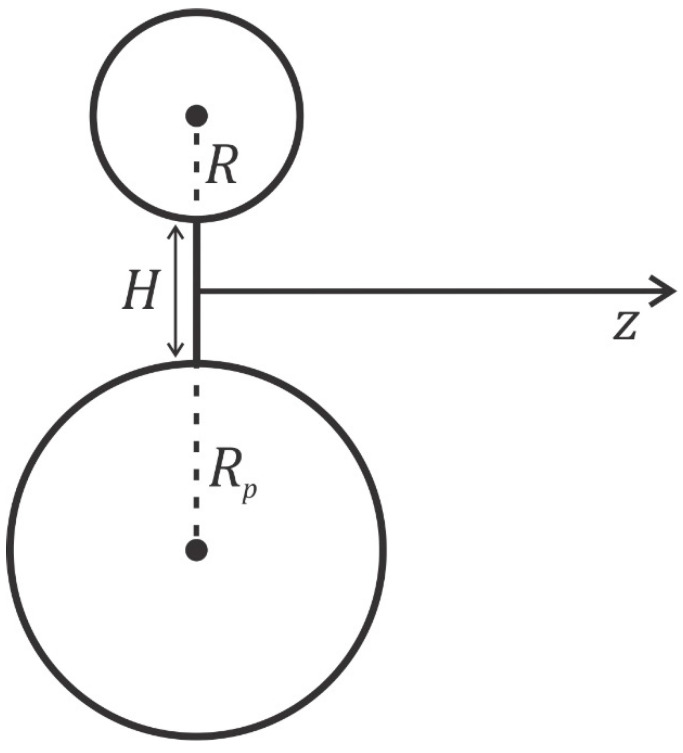
Schematic figure of two electrically charged spheres (vesicles) at the distance H with the radii $R_{p}$ and R.

As an example of application of modified LPB model, Figure 3 shows the electric potential distribution in the vicinity of two negatively charged spheres presented in the plane passing through the centres of both spheres (see also Figure 4). The spheres have uniformly distributed electric charges over the surface and are immersed in electrolyte solutions of monovalent ions. Figure 3 also shows the dependence of the magnitude of an electric field along the line starting at the midpoint between the two surfaces (z=0 point in Figure 4) in the direction perpendicular to the line that connects the centres of both spheres. If the radii of both spheres are equal, the electric field at the midpoint is zero (Figure 3a,b), while in the case of different radii of the spheres, the electric field at the midpoint is different from zero (Figure 3c). The electrostatic repulsion between two charged spherical vesicles may additionally facilitate the fission of vesicles in the process, as presented in Figure 1d,e. The method of calculation of osmotic pressure between two charged spherical vesicles as a function of the distance between them is presented in the next section.

### 3.2. Osmotic Pressure between Two Charged Surfaces within a Modified Langevin Poisson–Boltzmann Model

In the following, we derive, within the modified LPB theory, the expression for osmotic pressure between two charged planar surfaces (see Figure 2). First, we rearrange the modified LPB equation (Equation (2)) in planar geometry in the following form [55,100,104]:

(6) $$-\frac{d}{dx}\left[\varepsilon_{0}n^{2}\frac{d\phi }{dx}\right]-n_{0w}p_{0}\left(\frac{2+n^{2}}{3}\right)\frac{d}{dx}L\left(\gamma p_{0}E\left(x\right)\beta \right)+2e_{0}n_{0}\sinh\left(e_{0}\phi \beta \right)=0,$$

where we took into account Equation (1) for relative permittivity. Equation (6) is first multiplied by $\phi^{\prime }=d\phi /dx$ and then integrated to obtain [55,100]

(7) $$-\frac{1}{2}\varepsilon_{0}n^{2}E{\left(x\right)}^{2}+2n_{0}kT\cosh(-e_{0}\phi \beta )-n_{w}p_{0}(\frac{2+n^{2}}{3})E(x)L(\gamma p_{0}E(x)\beta )+\;+\left(\frac{2+n^{2}}{3}\right)\frac{n_{w}}{\gamma \beta }\ln\left[\frac{\sinh(\gamma p_{0}E\left(x\right)\beta )}{\gamma p_{0}E\left(x\right)\beta }\right]=K,$$

where the constant K in Equation (7) is the local pressure between the charged surfaces. Equation (7) is equivalent to the contact theorem. In order to obtain the net force per unit area between the charged surfaces [94], in the second step, we subtract the bulk values (outside the space between the charged surfaces) from the local osmotic pressure between the charged surfaces to obtain the expression for the osmotic pressure difference in the form $\Pi =\Pi_{inner}-\Pi_{bulk}$ [55,100]:

(8) $$\Pi =-\frac{1}{2}\varepsilon_{0}n^{2}E{\left(x\right)}^{2}+2n_{0}kT\left(\cosh\left(-e_{0}\phi \left(x\right)\beta \right)-1\right)-\;-n_{w}p_{0}\left(\frac{2+n^{2}}{3}\right)E\left(x\right)L\left(\gamma p_{0}E\left(x\right)\beta \right)+\left(\frac{2+n^{2}}{3}\right)\frac{n_{w}}{\gamma \beta }\ln\left[\frac{\sinh(\gamma p_{0}E\left(x\right)\beta )}{\gamma p_{0}E\left(x\right)\beta }\right],$$

where $\Pi_{bulk}=2n_{0}kT$. The osmotic pressure is constant everywhere in the solution between the charged plates (see also Figure 2). If both surfaces have equal surface charge densities ($\sigma_{1}=\sigma_{2}$), the electric field strength in the middle (x=H/2 in Figure 4) is zero (Figure 3); therefore, Equation (8) simplifies to the following form [55]:

(9) $$\Pi =2n_{0}kT\left(\cosh\left(-e_{0}\phi \left(x=H/2\right)\beta \right)-1\right).$$

Since at the midplane the electric field is zero and no force due to electric field acts on the ions, the entropic contribution to osmotic pressure is the most important (see also [94]).

**Figure 5:**
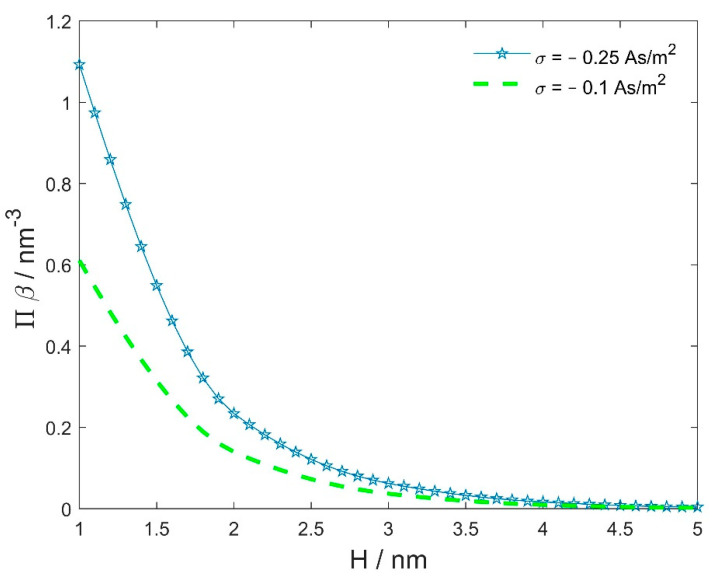
The calculated osmotic pressure between two negatively charged spherical vesicles of the same radius ($R_{p}=R=$ 10 nm) and same surface charge density as a function of the distance between their surfaces (*H*, see Figure 4). The values of the model parameters are $n_{0}/N_{A}=$ 0.15 mol/L, $n_{w}/N_{A}=$ 55 mol/L, and $p_{0}=$ 3.1 Debye.

Figure 5 shows the calculated osmotic pressure between two negatively charged spherical vesicles of the same radius as a function as the distance between them (H) (see also Figure 4), where Equation (9) for planar geometry was used to estimate the value of osmotic pressure. Note that, if the radii of the charged spheres are not equal, the electric field at the midpoint between the surfaces of the spheres is not zero (see Figure 3c). In this case, Equation (9) does not apply to estimating the osmotic pressure at the midpoint between the surfaces of the two spheres/vesicles and one should use the more general Equation (8).

**Figure 6:**
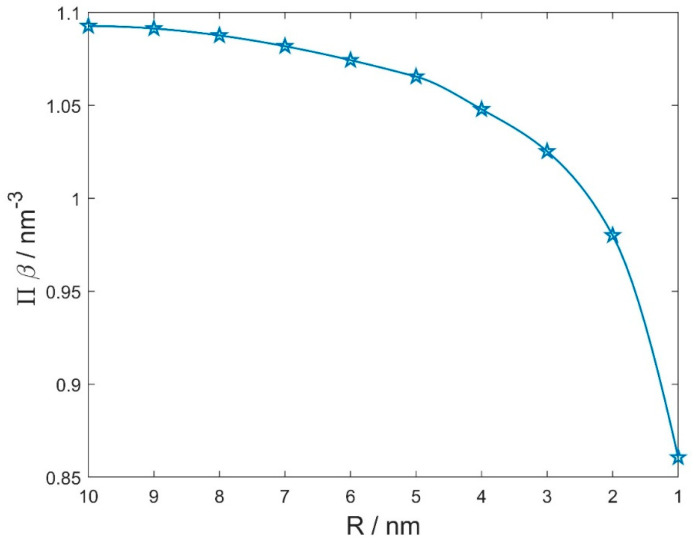
The calculated (net) osmotic pressure between two negatively charged neighbouring spheres/vesicles embedded in a electrolyte solution as a function of the radius of the smaller sphere/vesicle (R). The radius of the larger sphere/vesicle $R_{p}=$ 10 nm (see also Figure 4). The distance between the spheres/vesicles is H= 1 nm for all values of R. Both spheres have equal surface charge densities (σ= −0.25 As/m^2^). The value of the osmotic pressure for R= 10 nm corresponds to the case where both spheres/vesicles have the same radius (Figure 5). The values of other model parameters are the same as in Figure 5. Note that Equation (8) has limited validity for small values of R.

Figure 6 shows the calculated (net) osmotic pressure between the two charged spheres/vesicles in the electrolyte solution. The radius of the larger sphere/vesicles $R_{p}=$ 10 nm, while the radius of the smaller sphere/vesicle R (see Figure 4) varies between 1 nm and 10 nm. The radius 5 nm roughly corresponds to the dimension of the micelle, while radii smaller than 5 nm may correspond to charged organic or anorganic nanoparticles. The distance between the surfaces of the two neighbouring spheres/vesicles is fixed. It can bee seen in Figure 6 that the reduction in the radius of the smaller sphere/vesicle (R) from 10 nm to 5 nm (i.e., from the size of the vesicle to the size of the micelle) brings a rather small decrease in the osmotic pressure. The decrease in the size of the smaller sphere/vesicle becomes more pronounced only when the size is decreased down to the size of small nanoparticles. As we can see in Figure 5, the variation in the surface charge density of the spheres/vesicles has a stronger influence on the net osmotic pressure between the two neighbouring spheres/vesicles than the variation in the radii of the spheres/vesicles (Figure 6).

## 4. Conclusions

The degree of orientational order of the membrane constituents is strongly dependent on the membrane curvature and is influenced by both mechanical and geometrical/topological constraints. In the past, it was proposed that the orientational order of anisotropic membrane constituents may generate topological defects in the membrane regions of high anisotropic curvature at the points of frustration in ordered domains of membrane constituents.

It is shown in this work that the curvature induced topological antidefects may appear in the membrane necks as the membrane regions with the lowest degree of orientational ordering. Topological antidefects in the membrane necks connecting the beads of necklace-like membrane protrusions may induce fission of the protrusion into separated daughter vesicles as a result of the rupture of the necks, as shown in Figure 1c. The same mechanism may explain the fission of daughter vesicles, as shown in Figure 1e. As the main result of this work, we showed that the fission can be additionally facilitated by electric double-layer repulsion between the parent membrane and the membrane bud/vesicle, as shown in Figure 5.

## Data Availability

Not applicable.
